# Use of Point-of-care Ultrasound for Detection of Urethral Foreign Bodies: A Case Series

**DOI:** 10.5811/cpcem.41503

**Published:** 2025-10-24

**Authors:** Luca Tomasi, Michael Zampi, Michele Schroeder, Michael Cooper, Norah McIntyre

**Affiliations:** Baystate Medical Center, Department of Emergency Medicine, Springfield, Massachusetts

**Keywords:** foreign body, urethra, ultrasound, case series

## Abstract

**Introduction:**

Urethral foreign bodies are an uncommon presentation in the emergency department (ED) and can be difficult to assess and diagnose. There are examples in the literature of ultrasound detecting urethral foreign bodies. While not standard of practice, point-of-care ultrasound (POCUS) may be a useful tool for this unique pathology.

**Case Series:**

We describe three cases in which POCUS was used in the care of patients presenting with urethral foreign bodies. Ultrasound aided in diagnosis and helped facilitate further management.

**Conclusion:**

While urethral foreign bodies are relatively uncommon, they can lead to significant morbidity, which makes their prompt identification and treatment important. Ultrasound provides a rapid means of evaluation that allows the patient to stay under observation by ED staff while removing exposure to radiation or contrast.

## INTRODUCTION

Urethral foreign bodies are a relatively uncommon complaint in the emergency department (ED). Most cases tend to be in men, with a notable subset of pediatric patients.^1^ Common etiologies for these foreign body insertions are psychiatric, developmental, sexual gratification, and intoxication.^2^ Standard diagnostic modalities include plain radiography, computed tomography (CT), and cystoscopy. Ultrasound may hold advantages over alternative imaging, especially when behavioral problems make CT imaging difficult or when radiation is ideally avoided as in the pediatric population. Ultrasound can provide rapid evaluation of both radiolucent and radiopaque objects without the need for radiation or invasive procedure. Point-of-care ultrasound (POCUS) in the ED offers the additional benefit of rapid bedside diagnosis, which can then expedite treatment.

Point-of-care ultrasound has rarely been identified in the literature for detecting urethral foreign bodies.^3^,^4^ In this case series, we present three cases where POCUS was used in the ED to detect and localize urethral foreign bodies and help facilitate management. Our goal was to add to the growing body of literature about this novel diagnostic approach for urethral foreign bodies and to recommend the use of ultrasound in the workup of these uncommon presentations.

## CASE SERIES

### Case 1

**Image 1:**
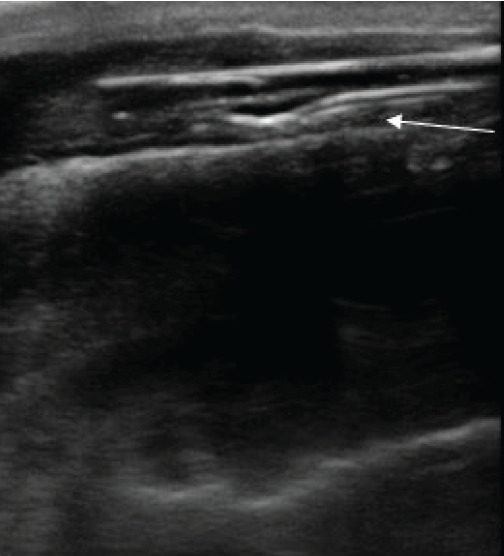
Longitudinal ultrasound view of the penis using a high-frequency linear probe. A hyperechoic linear object (arrow) is seen in the urethra, corresponding with the inserted foreign body.

A 22-year-old male with bipolar disorder and depression presented to our ED after intentional insertion of a piece of plastic into his urethra. This was not his first presentation; on history, he noted this was a “coping mechanism.” He complained of penile pain, dysuria, and hematuria, although he was able to urinate. The patient’s vital signs were normal. Physical exam revealed an unremarkable abdominal exam. He had tenderness to palpation along the shaft of the penis with a palpable foreign body, but nothing was visualized at the urethral meatus. Plain radiography was interpreted as normal with no visualized foreign body. Lab work and urinalysis were also normal. Clinicians using POCUS were able to visualize a linear hyperechoic object within the urethra (Image 1). Urology was consulted and took the patient to the operating room (OR) for removal with cystoscopy. In the OR a rolled-up piece of plastic was identified three centimeters into the urethra and was successfully removed. The patient was discharged without complication the following day.

### Case 2

A 60-year-old male with a history of depression, anxiety, post-traumatic stress disorder, insomnia, and self-harm with past foreign body insertion into the urethra presented for foreign body obstructing his urethra. Approximately nine hours prior to evaluation by emergency physicians, the patient described having an episode of anger, subsequently inserting five baby carrots into his penis. He stated that several of the baby carrots came out of the urethra but estimated two remained inserted. He attempted removal with chopsticks but was unsuccessful. Since inserting the foreign bodies, the patient noted pain and difficulty urinating. While he had inserted foreign bodies into his urethra in the past, he had never required evaluation in the ED or urology consultation. In the ED, the patient was hypertensive to 180/96 millimeters of mercury; otherwise vital signs were stable. He was anxious. There were no findings on external genitourinary exam.

*CPC-EM Capsule*

What do we already know about this clinical entity?

*Urethral foreign bodies rarely present to the ED but can result in significant morbidity. Diagnostic modalities include CT, plain radiography, and cystoscopy*.

What makes this presentation of disease reportable?

*Ultrasound is not the standard imaging modality to diagnose urethral foreign bodies. We report on its use to detect foreign bodies and help facilitate management*.

What is the major learning point?

*Point-of-care ultrasound provides a rapid means of evaluating patients with urethral foreign bodies*.

How might this improve emergency medicine practice?

*Use of ultrasound to diagnose urethral foreign bodies may result in less exposure to radiation, lower cost, and expedited care*.

**Image 2:**
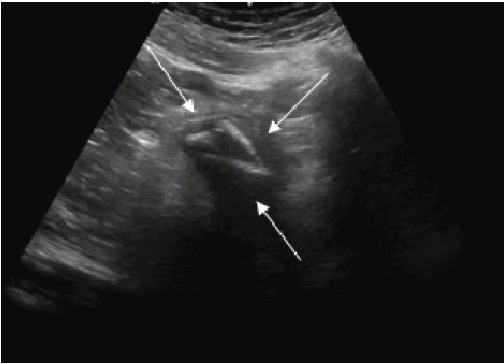
Sagittal ultrasound view of the bladder using a curvilinear probe. Three hyperechoic foreign bodies (arrows) are seen in the bladder.

Bedside ultrasound identified three linear hyperechoic objects in the bladder (Image 2), with none seen in the urethra. This was followed by a CT, which confirmed the findings; three foreign bodies were retained in the bladder. Urology was consulted, and the patient was admitted to the hospital. Urology performed a cystoscopy with open cystotomy, removed the foreign bodies, and placed a Jackson-Pratt (JP) drain and Foley catheter. The hospital course was complicated by traumatic damage to the urethra, small extravasation from the bladder, and *Enterococcus faecalis* urinary tract infection. Infectious disease and psychiatry were consulted. The patient completed a course of antibiotics, had the JP drain and Foley catheter removed, and was discharged on hospital day 20.

### Case 3

**Image 3:**
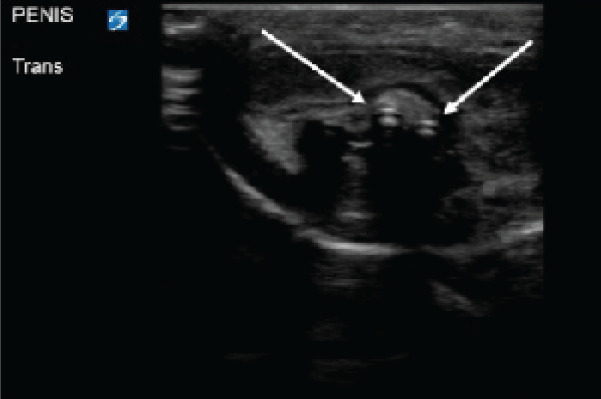
Transverse ultrasound view of the penis using a linear probe. A hyperechoic foreign body (white arrows) seen in the urethra, with evidence of looping of the object.

An 11-year-old male presented with the chief complaint of foreign body inserted into his urethra. The patient had inserted a braided USB-type cord without hub attachments into his urethra. He had similarly inserted foreign bodies into his urethra in the past but stated, “This is the first time I couldn’t get it out.” On arrival the patient was in no acute distress. Genitourinary exam was significant for a single, braided cord protruding from the urethra. Neither the patient nor the emergency physicians were able to extract the cord with gentle traction. Using POCUS, the clinicians identified the cord in the bladder, as well as evidence that the cord had looped within the urethra (Image 3). Urology was consulted, and a urologist evaluated the patient at bedside. The patient underwent moderate sedation with ketamine. The cord had in fact looped once in the urethra. The cord was extracted by the urologist, with overall no complications besides the looping in the urethra. The patient had residual scant hematuria that resolved at the time of outpatient urology follow-up.

## DISCUSSION

While self-insertion of urethral foreign bodies is a rare presentation to the ED, it is an issue requiring rapid assessment and intervention. A single-center study found that of almost 18,000 admitted patients in the six years prior to its publication, only 10 presented with urethral foreign bodies, representing 0.055% of the admitted population.^2^ Despite the low incidence of this presentation, there is significant morbidity including infection and loss of function, which makes prompt evaluation and care imperative for these patients.^5^ This is even more important when considering that many of these patients may present delayed due to embarrassment or concomitant psychiatric illness.^1^ While urethral foreign bodies can often be diagnosed with history and exam alone, CT and plain radiography are often used as well. This is particularly common when specific information such as location is unknown or if history and exam are inadequate. Cystoscopy is often necessary for removal of these objects, and less frequently open surgery, if the object cannot be removed endoscopically.

Point-of-care ultrasound provides multiple benefits while avoiding many of the drawbacks of more standard evaluation. Plain radiography can be helpful and performed at the bedside if needed; however, it is only useful if the object in question is radiopaque. As our case series demonstrates, POCUS is capable of viewing objects that are both radiopaque and radiolucent. Additionally, plain radiography shows a two-dimensional picture while POCUS can be used to obtain views in multiple planes to better map out an object’s shape, location, and orientation. While CT provides a detailed, three-dimensional picture, it requires patient cooperation, as well as a substantial exposure to radiation. This is potentially exacerbated by the fact that some of these patients may have similar repeated presentations, especially if being driven by a behavioral problem or sexual gratification. Additional considerations are cost and time to obtain the imaging, often requiring transporting the patient to another area for imaging.

While POCUS does not provide the same level of detail as a CT, it does allow visualization of the object as well as provide information regarding shape, orientation, and location. Point-of-care ultrasound images can be obtained quickly, and the images are interpreted by the emergency physician, expediting imaging results. Additional benefits of ultrasound include avoiding radiation, lower cost, and not requiring the patient to remain still. Point-of-care ultrasound certainly has a role as a diagnostic tool for urethral foreign bodies, but it is rarely used in this capacity.

In this paper, we suggest a novel diagnostic approach using POCUS. Current barriers to using POCUS for this chief complaint likely include not realizing ultrasound can play a role, lack of confidence to obtain adequate images, and inability to interpret images. Incorporating additional training into existing ultrasound curricula would likely increase familiarity and comfort with this specific modality, in addition to reducing the risk of inappropriate interpretation of imaging. Medicolegal concerns for the incorporation of POCUS in such cases may be mitigated by establishing diagnostic protocols, as well as clear documentation of findings. Additional case series and studies focusing on this diagnostic approach could lead to POCUS as the standard of care in initial ED evaluations for urethral foreign bodies.

## CONCLUSION

Urethral foreign bodies are an uncommon presentation to the emergency department, often requiring imaging modalities to provide necessary information to guide management. This paper describes three cases where POCUS was used in the diagnosis of urethral foreign bodies and helped facilitate management. Ultrasound has advantages over alternative imaging and, as demonstrated here, has proven to be useful. Patient care may benefit from more routine use of this tool in the evaluation of patients with urethral foreign bodies.
